# Serum protein profile in systemic-onset juvenile idiopathic arthritis differentiates response versus nonresponse to therapy

**DOI:** 10.1186/ar1723

**Published:** 2005-04-04

**Authors:** Takako Miyamae, David E Malehorn, Bonnie Lemster, Masaaki Mori, Tomoyuki Imagawa, Shumpei Yokota, William L Bigbee, Manda Welsh, Klaus Klarskov, Norihiro Nishomoto, Abbe N Vallejo, Raphael Hirsch

**Affiliations:** 1 Division of Rheumatology, Children's Hospital of Pittsburgh, Department of Pediatrics, University of Pittsburgh School of Medicine, Pittsburgh, PA 15213; 2 University of Pittsburgh Cancer Institute, University of Pittsburgh School of Medicine, Pittsburgh, PA 15213; 3 Department of Pediatrics, Yokohama City University School of Medicine, Yokohama, Japan; 4 Départment de Phamacologie, Faculté de Medicine, Université de Sherbrooke, Québec, Canada; 5 Osaka University, Osaka, Japan

## Abstract

Systemic-onset juvenile idiopathic arthritis (SJIA) is a disease of unknown etiology with an unpredictable response to treatment. We examined two groups of patients to determine whether there are serum protein profiles reflective of active disease and predictive of response to therapy. The first group (*n *= 8) responded to conventional therapy. The second group (*n *= 15) responded to an experimental antibody to the IL-6 receptor (MRA). Paired sera from each patient were analyzed before and after treatment, using surface-enhanced laser desorption/ionization time-of-flight mass spectrometry (SELDI-TOF MS). Despite the small number of patients, highly significant and consistent differences were observed before and after response to therapy in all patients. Of 282 spectral peaks identified, 23 had mean signal intensities significantly different (*P *< 0.001) before treatment and after response to treatment. The majority of these differences were observed regardless of whether patients responded to conventional therapy or to MRA. These peaks represent potential biomarkers of active disease. One such peak was identified as serum amyloid A, a known acute-phase reactant in SJIA, validating the SELDI-TOF MS platform as a useful technology in this context. Finally, profiles from serum samples obtained at the time of active disease were compared between the two patient groups. Nine peaks had mean signal intensities significantly different (*P *< 0.001) between active disease in patients who responded to conventional therapy and in patients who failed to respond, suggesting a possible profile predictive of response. Collectively, these data demonstrate the presence of serum proteomic profiles in SJIA that are reflective of active disease and suggest the feasibility of using the SELDI-TOF MS platform used as a tool for proteomic profiling and discovery of novel biomarkers in autoimmune diseases.

## Introduction

Systemic-onset juvenile idiopathic arthritis (SJIA) is a form of childhood arthritis of unknown etiology, characterized by systemic features in addition to arthritis, including spiking fever, erythematous rash, articular involvement, and other, visceral manifestations [1]. Its clinical course is associated with changes in the levels of several serum proteins, including IL-6 [2]. Over half of children with SJIA eventually recover almost completely [3]. The other half have severe, unremitting arthritis, poorly responsive to conventional therapy, leading to poor functional outcome and substantial morbidity [4]. In view of the heterogeneity of clinical disease manifestations and the unpredictability of treatment responses in SJIA, there would be great clinical benefit in the discovery of biomarkers reflective of disease activity and predictive of response to therapy.

Proteomics, or protein pattern analysis, is the characterization and quantitation of proteins in tissues and body fluids [5]. Proteomic methods can be used to compare protein expression patterns between disease states. Although two-dimensional gel electrophoresis has been the primary technique in conventional proteomic analysis, it is relatively insensitive to proteins of low abundance and below 10 kDa in mass, is labor intensive, and has low throughput. A more recent technology known as surface-enhanced laser desorption/ionization time-of-flight mass spectrometry (SELDI-TOF MS), a derivative of conventional matrix-associated laser desorption/ionization time-of-flight mass spectrometry (MALDI-TOF MS), involves the application of a biologic sample, such as serum, to a protein-binding chip [6]. The chip is irradiated with a laser, resulting in ionization of the adherent molecules. The ions travel through a vacuum tube and their mass-to-charge ratios are calculated from their time of flight through the vacuum chamber. The technology is high throughput, rapid, and sensitive and provides a profile of low-molecular-weight peptides and proteins within a complex mixture such as serum.

SELDI-TOF MS does not directly identify specific proteins. It has been used to differentiate disease states from nondisease states by analysis of protein profiles in sera. Examples include the differentiation of neoplastic from non-neoplastic breast masses [7], prognostic and diagnostic classification of breast cancer [8], neoplastic versus non-neoplastic disease of the ovary [9], and prostate cancer from both men with benign hyperplasia and healthy men [10]. SELDI-TOF MS has also been used for the discovery of disease-related biomarkers in sera. Examples include detection of serum amyloid α in patients with renal cancer [11] and the quantitation of prostate-specific membrane antigen in prostate cancer [12].

The present study was designed to determine whether there are serum proteomic profiles in SJIA that are reflective of active disease and predictive of response to therapy, as well as to determine whether SELDI-TOF MS could be used as a tool for proteomic profiling and for discovery of novel biomarkers of SJIA.

## Materials and methods

### Patients and study subjects

Banked sera from 23 patients (14 boys, 9 girls) with SJIA according to the criteria established by the International League of Association for Rheumatology [13] were obtained from the Department of Pediatrics, Yokohama City University School of Medicine, Yokohama, Japan. All the patients were Asian and their mean age at the start of the study was 7.25 ± 0.92 years. Eight of them had obtained a clinical response to conventional therapy. Clinical response was defined as the absence of fever rash, hepatosplenomegaly, and arthritis for at least 3 months, accompanied by normalization of serum C-reactive protein. Briefly, conventional therapy consisted of three doses of intravenous methylprednisolone (30 mg/kg per day) or oral prednisolone (1 to 2 mg/kg), followed by nonsteroidal anti-inflammatory drugs (NSAIDs) and a tapering dose of oral prednisolone. In addition, methotrexate (2.5 to 5 mg/m^2 ^per week orally) was used in three patients and cyclosporin (5 mg/kg per day orally) in two. The mean period from acute status to complete clinical response was 27.7 ± 14.6 months. Fifteen patients who had inadequate response to the above therapy, as well as to the addition of azathioprine (five patients), mizoribine (five patients), sulfasalazine (two patients), or plasma exchange (three patients), had been administered humanized anti-IL-6 receptor antibody (MRA; Chugai Pharmaceuticals, a subsidiary of Roche Pharmaceuticals). All 15 patients had a clinical response to MRA. The mean period from acute status to clinical response was 11.2 ± 5.1 months. Pretreatment sera were collected before starting conventional treatment or giving the initial dose of MRA. Post-treatment sera were collected 2 to 3 months after patients achieved a clinical response. Ethical approval for this study was granted by Yokohama University. The study was approved by both Chugai Pharmaceuticals and Roche Pharmaceuticals.

### SELDI-TOF MS

Serum samples were thawed on ice, denatured, and processed in duplicate on IMAC-3 (immobilized metal affinity capture) copper ProteinChip^® ^Arrays (Ciphergen Biosystems, Fremont, CA, USA). ProteinChips were loaded, processed, and prepared for mass spectrometry using a Biomek2000 liquid handling robot (Beckman-Coulter, Fullerton, CA, USA) and optimized for reproducibility using validated protocols. ProteinChips were read in a PBSIIc mass spectrometer (Ciphergen) with mass deflection at 1 kDa and time-lag focusing. The resulting mass spectra were examined between *m*/*z *values of 2 and 100 kDa for quantitative comparison of identifiable peak features. The parameters used for spectral preprocessing and peak selection were: external calibration (seven peptide calibrants, 1 to 7 kDa, Ciphergen), baseline subtraction by 8 × expected peak width and smoothing, filtering by average using 0.2 expected peak width, noise defined over 1500 Da, normalization by total ion current (TIC) over 1500 Da, peaks detected over 2000 kDa by centroid mass. Weak spectra were excluded from analysis if the normalization factor exceeded 2 standard deviations above the mean normalization factor.

### Statistical analysis

Peak clustering among sample groups was performed with the Biomarker Wizard (Ciphergen) tool, with a peak detection threshold of 5 for signal-to-noise ratio, and mass tolerance of 0.3%, for any peak appearing in at least 5% of experimental spectra being compared. The Biomarker Wizard compares the mean intensity of peak clusters, by sample group, using the nonparametric Mann–Whitney *U *test (two–way comparisons) and generates *P *values that reflect probabilities that mean peak intensities at a given *m*/*z *value differ by random chance. The intensity values of the automatically clustered peaks (averaged between technical replicates of each sample) were used in classification tree analysis (CART) using Ciphergen's Biomarker Patterns Software. This supervised learning process uses cross-validation to optimize the minimization of classification error.

### Immunoprecipitation of SAA

A pooled sample from four sera taken before conventional treatment was incubated with either Protein A–Sepharose beads alone or Protein A–Sepharose beads bound with 100 μg of anti-SAA antibody (Anogen, Yes Biotech Laboratories, Mississauga, ON, Canada). After immunoprecipitation, the depleted serum was subjected to SELDI-TOF MS.

### LC-ESI-MS/MS-TOF analysis

Protein identification by MS was carried out as previously described [14]. Briefly, serum samples were subjected to immunoprecipitation with anti- SAA or with an IgG isotype control. Immunoprecipitates were washed extensively in phosphate-buffered serum and centrifuged, and the pellet was sonicated for 10 min in 8 M urea/400 mM NH_4_HCO_3_. The supernatant was diluted in water to a final concentration of 2 M urea/100 mM NH_4_HCO_3 _and digested overnight at 37°C with 1 μg trypsin. The tryptic digest was subjected to nano-LC-ESI-MS/MS analysis that was performed on a Q-TOF-2™ (Waters, Milford, MA, USA), coupled on line to a CapLC system equipped with three separate syringe pump modules, an auto injector, a 10-port valve and a 250-μm (inner diameter) × 1-mm pre-column. Separations were performed on a 7-cm × 75-μm (inner diameter) capillary column. Both columns were packed with Microsorb C18 (Varian, Mississauga, ON, Canada) reverse-phase material. Peptides were eluted at a flow rate of 0.25 μl/min with the following linear gradient of solvent B (80% aqueous acetonitrile with 10% isopropanol and 0.2% formic acid) in solvent A: from 0 to 60% B in 40 min, to 90% B in 7 min, and to 10% B in 8 min. Spectra were acquired in auto MS/MS mode conducted using survey scans to choose up to three precursor ions. Collision energies were selected automatically as a function of *m*/*z *value and charge state. The Q-TOF mass spectrometer was calibrated by infusing a solution of either NaI containing a small amount of cesium ion dissolved in 50% aqueous isopropanol (0.2 μg/μl) or Glu-fibrinopeptide B (1 pmol/μl) dissolved in 30% aqueous acetonitrile containing 0.2% formic acid. Protein identification was performed using the MASCOT search program (Matrix Science Limited, ) and the NCBI (National Center for Biotechnology Information) (Bethesda, MD, USA) protein database.

## Results

### Protein profiling by SELDI-TOF MS reveals distinct patterns differentiating active from well-controlled SJIA

**Figure 1:**
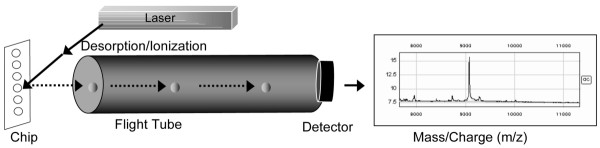
SELDI-TOF MS technique. Serum samples are spotted onto IMAC-3 copper chips^® ^(Ciphergen Biosystems). The chip is irradiated with a laser, resulting in ionization of the adherent molecules. The ions travel through a vacuum tube and their mass-to-charge ratios are calculated from their time of flight through the vacuum chamber. IMAC-3, immobilized metal affinity capture; SELDI-TOF MS, surface-enhanced laser desorption/ionization time-of-flight mass spectrometry.

**Figure 2:**
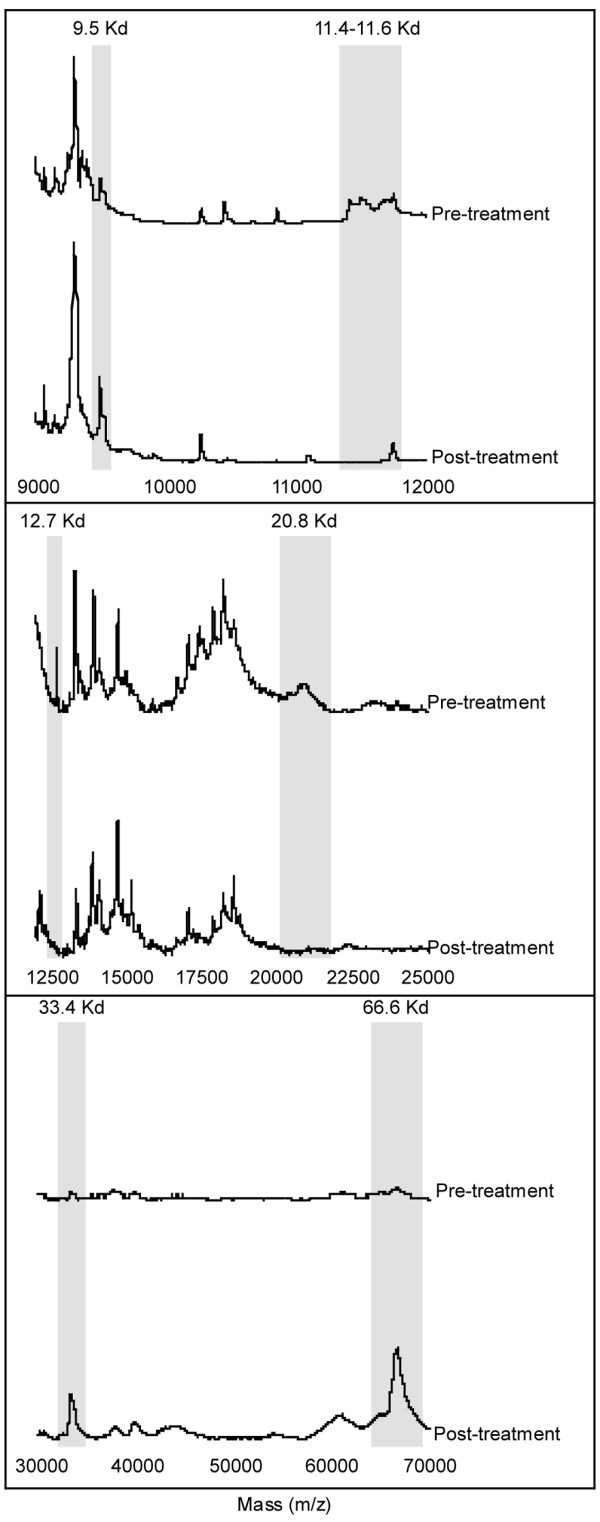
SELDI-TOF MS profiles for patients with SJIA treated conventionally. Six serum protein peaks can be clearly seen to have changed after conventional therapy. The profiles of a representative patient are shown here. Visually distinct peaks that were clearly different between pre- and post-treatment paired samples upon visual inspection of the profiles (in bold type in Table 1) are outlined in grey. Pre- and post-treatment spectra are shown on the same intensity scale in each frame. SELDI-TOF MS, surface-enhanced laser desorption/ionization time-of-flight mass spectrometry SJIA, systemic juvenile idiopathic arthritis.

To determine whether SELDI-TOF MS could be a valuable tool for analyzing serum protein profiles in autoimmunity, we chose SJIA as a test model, since the disease has systemic features, in addition to arthritis, likely to be reflected in the serum. Paired sera were available from patients who had been followed up for a mean of 24.4 ± 6.9 months. The availability of paired sera from each patient allowed for longitudinal comparison and substantially reduced sample variability between the two groups. Sera from eight patients with SJIA who responded to conventional therapy (therapy and definition of clinical response are described in detail in the Materials and methods section) were analyzed before and after therapy by SELDI-TOF MS using Ciphergen IMAC-3 (immobilized metal affinity capture) copper chips (Fig. 1). All samples were run in duplicate. Table 1 shows the most significant differences between mass spectra of sera before and after conventional therapy. Only variables with nonparametric *P *values of <0.001 are given. Of 282 spectral peaks identified, 23 had mean signal intensities that were significantly different (*P *< 0.001) before and after response to treatment. These peaks represent potential biomarkers of active disease. We next performed a similar analysis on paired sera from 15 patients who had failed conventional therapy but responded to an experimental antibody to the IL-6 receptor (MRA) [15]. These sera were obtained after failure of conventional therapy. Pre- and post-MRA sera revealed similar profiles to those observed in the pre- and post-conventional therapy group. Thus, substantial consistency was observed in protein profiles, regardless of whether patients with active disease responded to conventional therapy or to MRA. Eight of the differentially expressed peaks represent prominent, visually distinct spectral features. These peaks are represented in Table 1 in bold, along with the number of paired patient sera in which each peak was differentially expressed by visual inspection of the spectra. Representative examples of these peaks are shown in Fig. 2.

To determine the usefulness of the profiles in classifying active versus controlled SJIA, the data were subjected to CART (Biomarker Patterns Software, Ciphergen) analysis. This sample classification method is designed, through multivariate analysis, to construct classification trees recognizing a complex pattern of multiple peak intensities. The method is ideally suited for sample sets large enough to permit cross-validation internal to the 'training' data, but also the segregation of additional unused data as a validation or 'testing' set. On these relatively small sample sets, CART was used in training mode primarily as a data exploration tool. Whether using the training set as the MRA group, or as the conventional treatment group, the CART analysis returned simple classification trees consisting of one primary splitter, either 11.4 kDa or 11.6 kDa (*m*/*z*). The primary splitter at 11493 kDa correctly identified 13 of 14 pretreatment and 14 of 15 post-MRA treatment samples when conventional treatment was used as the training set. When MRA treatment was used as the training set, all of the pre- and post-conventional treatment samples were correctly identified as either pretreatment or post-treatment. The distinction between these samples by CART registered at the most extreme level of significance the program is capable of indicating. Even when forced to ignore the mass spectrum peaks at 11493 or 11650 Da, the CART program was able to effectively discriminate, using secondary peaks derived from them (at half these *m*/*z *values; attributed to doubly protonated species). This robust classification surpasses the performance of any other sample set being profiled and analyzed by this and several other statistical methods at this institution (data not shown).

### Identification of serum amyloid A from SELDI-TOF MS mass spectra

**Figure 3:**
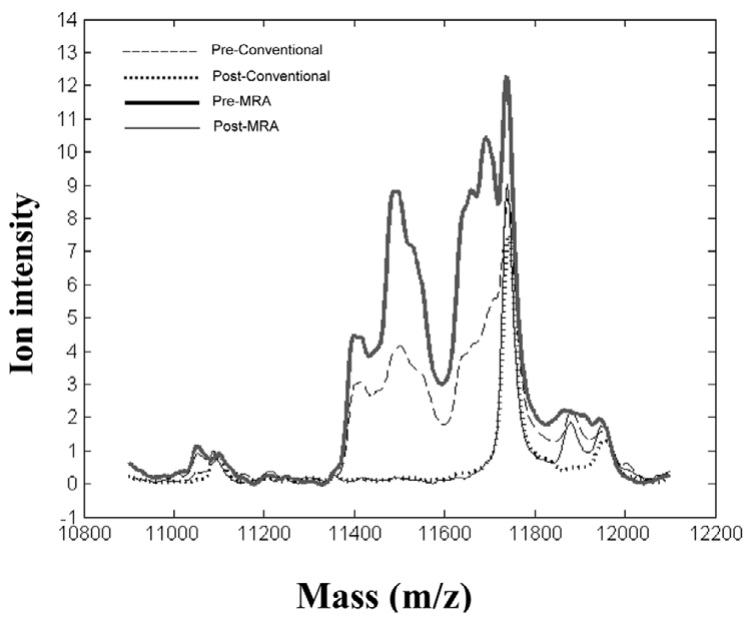
SELDI-TOF MS profiles for patients with SJIA before and after conventional or MRA treatment. Sera taken before conventional and MRA treatment show similar patterns that are distinct from the post-treatment profiles. Mean spectra of all patients are shown in the 11- to 12-kDa *m*/*z *range. Means are compiled from 8 samples before and after conventional therapy and 15 samples before and after MRA therapy, each sample run in duplicate. Spectra were preprocessed as described in the Materials and methods section. MRA, humanized anti-IL-6 receptor monoclonal antibody; SELDI-TOF MS, surface-enhanced laser desorption/ionization time-of-flight mass spectrometry SJIA, systemic juvenile idiopathic arthritis.

**Figure 4:**
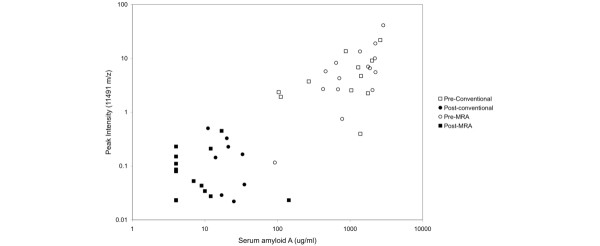
Peak intensities of the 11.6-kDa *m*/*z *SELDI peak in serum after MRA treatment in SJIA. The peak intensities correlated with the SAA titers measured by latex agglutination. MRA, humanized anti-IL-6 receptor monoclonal antibody; SAA, serum amyloid A; SELDI, surface-enhanced laser desorption SJIA, systemic juvenile idiopathic arthritis.

**Figure 5:**
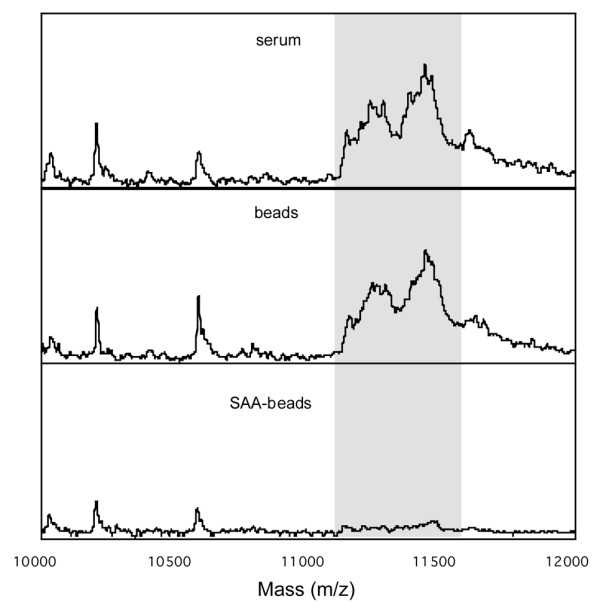
Immunoprecipitation of SAA in SJIA, resulting in loss of the 11.4- to 11.6-kDA peak cluster. A pooled sample from four sera before conventional treatment (top panel) was incubated with either Protein A–Sepharose beads alone (middle panel) or Protein A–Sepharose beads bound with anti-SAA antibody (lower panel). After immunoprecipitation, the depleted serum was subjected to SELDI-TOF MS. The 11.4-to 11.6-kDa *m*/*z *peak cluster is shown in grey. MRA, humanized anti-IL-6 receptor monoclonal antibody; SAA, serum amyloid A; SELDI-TOF MS, surface-enhanced laser desorption/ionization time-of-flight mass spectrometry; SJIA, systemic-onset juvenile idiopathic arthritis.

A prominent group of peaks within the range 11.4 to 11.7 kDa *m*/*z *strongly distinguished the pre- and post-treatment samples (Fig. 3). The post-treatment groups showed an apparently single *m*/*z *peak at 11.75 kDa, which was also routinely observed in pooled reference sera from healthy adults (data not shown). A previous study in nasopharyngeal cancer, using the same SELDI-TOF technique and IMAC3 copper chip, identified two biomarkers, of 11.6 and 11.8 kDa, as serum amyloid A (SAA) [16]. Since SAA is a known biomarker of active SJIA [17], we compared the intensity of the 11.6-kDa peak with SAA levels in the sera, as determined by latex agglutination. As shown in Fig. 4, a strong correlation was observed (*R*^2 ^= 0.74), suggesting that the 11.6-kDa peak might represent SAA. To further investigate the biochemical identity of this peak, serum containing high levels of the 11.6-kDa peak was subjected to immunoprecipitation using anti-SSA antibody bound to Protein A–Sepharose beads. As shown in Fig. 5, after immunoprecipitation and SELDI analysis, the 11.4- and 11.6-kDa peaks were markedly diminished. To confirm the identity of the immunoprecipitated protein, anti-SAA precipitates were digested with trypsin and subjected to η-scale liquid chromatography electrospray ionization tandem mass spectrometry time-of-flight (LC-ESI-MC/MS-TOF) analysis. Over 90 peptide ions were examined and only 2 proteins were identified, including immunoglobulin and SAA. The SAA peptide ions represented approximately 51% of the SAA sequence.

### Protein profiling by SELDI-TOF MS reveals patterns differentiating the responding from the nonresponding SJIA group

**Figure 6:**
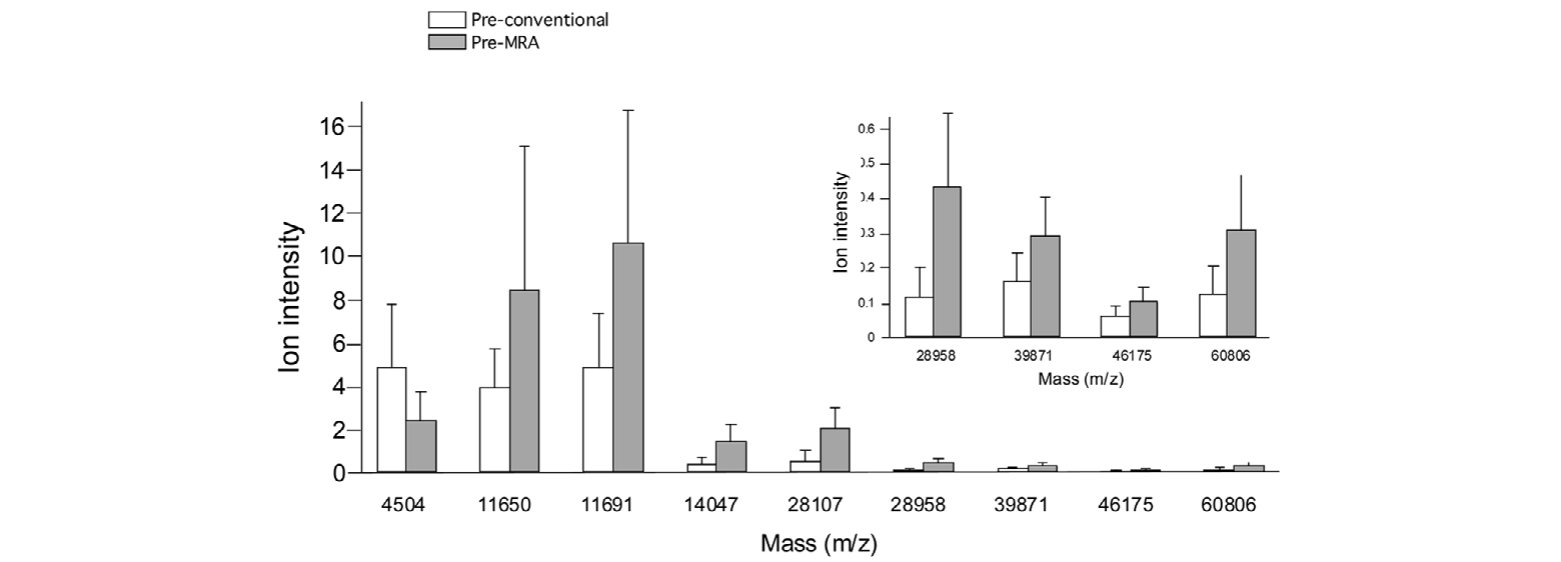
Differences between mass spectra of sera before and after MRA treatment for SJIA. Sera were taken before and after treatment with MRA from patients for whom conventional therapy had failed. The high significance of these differences suggests a profile predictive of response to conventional therapy. The values represent mean intensities. The *P *values of these univariate comparisons are given in Table 2. Error bars represent standard deviations. MRA, humanized anti-IL-6 receptor monoclonal antibody; SJIA, systemic-onset juvenile idiopathic arthritis.

The above data, using paired sera, demonstrate the ability of SELDI-TOF MS to identify biomarkers of active disease, as exemplified by the identification of SAA. A long-term goal is to predict clinical outcome, based on protein profiles present in the serum early in the disease course. To begin to approach this challenge, we compared the pretreatment serum profiles of the 8 patients who responded to conventional therapy with those from the 15 patients who responded poorly to conventional therapy. Similar to the preceding analysis, the latter samples were obtained after failure of conventional therapy and before MRA treatment, when the patients still had active disease. In this initial exploratory study, the number of available samples was too small for definitive conclusions; however, several interesting trends were apparent. Several highly significant differences were observed in the mass spectra of these sera, as shown in Table 2 and Fig. 6. These peaks may represent a profile predictive of response to conventional therapy. Alternatively, they could represent the effects of conventional therapy or differences between early versus long-standing disease. A number of peaks overlap with regions observed in Table 1, including the region of SAA (11.6 kDa) as well as 4504 kDa and 28 kDa.

**Figure 7:**
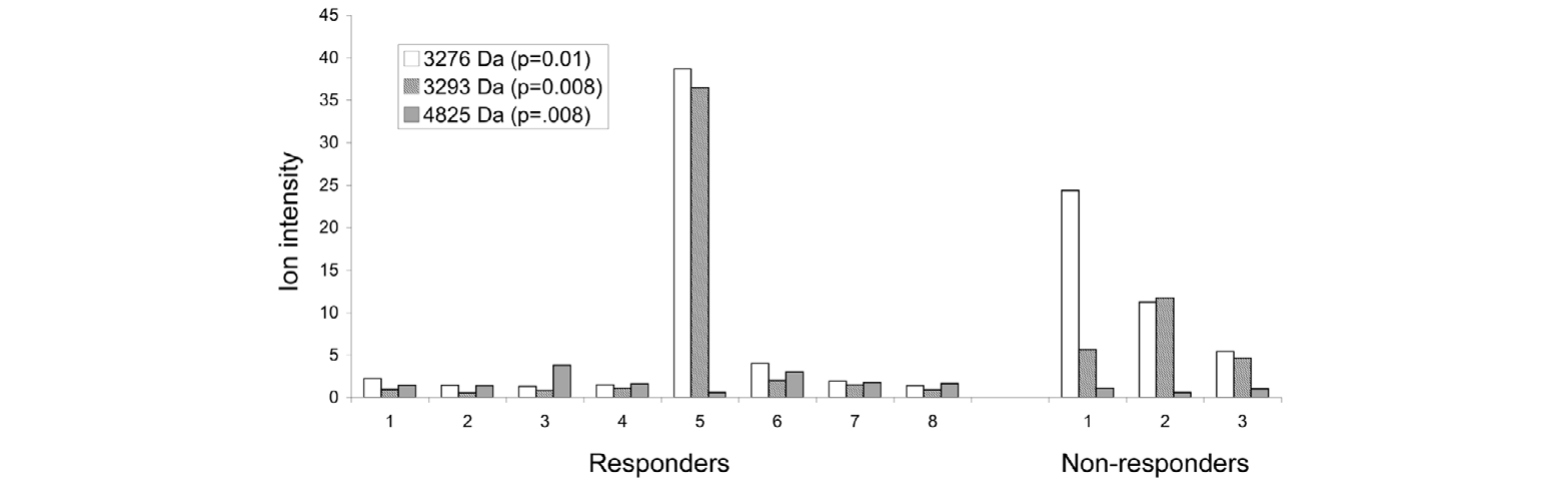
Serum proteins in SJIA patients according to whether they responded to conventional therapy. Three most significant differences distinguishing between pretreatment samples from conventional therapy responders (*n *= 8) and those from nonresponders (*n *= 3), suggesting a profile predictive of response to conventional therapy. The averaged peak intensity is shown for the eight pretreatment 'responder' patient samples (left panel) compared with the corresponding intensities of those same three peaks from the three pretreatment 'nonresponder' patient samples (right panel). SJIA, systemic-onset juvenile idiopathic arthritis.

We were fortunate to have pre-conventional treatment sera available from 3 of the 15 patients who responded poorly to conventional therapy and went on to receive MRA. These three pretreatment sera were compared with the pretreatment sera of the eight patients who responded to conventional therapy. Three peaks of interest were observed. As shown in Fig. 7, all the nonresponders had lower values for the 4825-Da feature and higher values for the 3276-Da and 3293-Da peaks than did the responders, with the exception of a single outlier sample. However the sample size is small and this observation needs further validation in a larger clinical cases series; this putative signature of nonresponse may be susceptible to statistical overfitting, even at this level of analysis.

## Discussion

Current diagnostic techniques for rheumatic diseases are based on clinical presentation and nonspecific serum markers. Because the phenotype of a rheumatic disease such as SJIA is largely dependent on proteins, the present study was designed to determine whether serum protein expression profiling with SELDI-TOF MS could be used to search for new molecular diagnostic biomarkers and potential therapeutic targets. This approach has theoretical advantages over other modalities used to identify differentially expressed proteins. SELDI-TOF MS analysis is capable of detecting small amounts of protein, hence the potential to detect proteins of relatively low abundance with affinity for the ProteinChip surface. The technique is high throughput, allowing detection of hundreds of species in a single sample, and is capable of analyzing large number of samples. The data presented here show that it is possible to generate mass spectrometry protein expression profiles from serum that can differentiate active versus controlled SJIA.

Some of the difficulties inherent in gene or protein expression profiling in human disease include accounting for the genetic and environmental variability between patients and the potential for detecting chance associations when measuring large numbers of proteins or genes. The use of paired serum samples from individual patients in the present study removes most of these variables and makes it likely that the changes in protein profiles after successful treatment reflect the disease state rather than confounding variables. We found a surprising degree of consistency in the relative abundance of a number of serum proteins in ill versus well patients. The clear distinction in the levels of these various ionic species between these sample groups permits a robust classification based on simple thresholding on any one of a number of possible variables.

One disadvantage of the SELDI-TOF MS technology is that protein sequences, and thus specific identifications, are not obtained, requiring further biochemical/mass spectrometry analysis to identify differentially-expressed proteins. A recent study using two-dimensional gels and MALDI-TOF MS analysis of plasma and synovial fluids from patients with rheumatoid arthritis or osteoarthritis also revealed the presence of SAA in samples from rheumatoid arthritis but not osteoarthritis [18]. Although SELDI-TOF MS is not directly quantitative, it can detect changes in the relative abundance of proteins in a manner that compares favorably to quantitative methods such as latex agglutination or enzyme-linked immunosorbent assay. Identification of SAA by SELDI-TOF MS helps validate our experimental approach, since SAA is a known marker of active SJIA.

Although we were able to identify SAA by further analysis, there were many other peaks observed in the serum profiles that have yet to be explored or identified. The 66.6-kDa and 33.4-kDa peaks most likely represent serum albumin and its doubly protonated form, as they are the correct mass and they increase after response to therapy, reflective of the known rise in serum albumin levels in these patients (data not shown). Identification of the other peaks is currently being investigated and may yield novel information on the pathophysiology of SJIA. Furthermore, the proteins observed represent only a fraction of those present in the serum. We observed only a subset of relatively high-abundance proteins, limited by their concentration in the serum, their affinity with the copper matrix of the IMAC ProteinChip, and the relative desorption/ionization efficiencies of each protein. In addition, the fact that the mass spectra generated in this study were from unfractionated sera is likely to obscure many protein species that might otherwise be detectable in the absence of high-abundance serum proteins. Refinement of the methodologies for processing serum samples, including initial depletion of high-abundance proteins, is likely to substantially increase the information that can be derived from the resulting profiles.

There are likely many more subtypes of the group of diseases known collectively as idiopathic arthritis than have as yet been defined by clinical criteria. The ability to differentiate uncontrolled from controlled SJIA by serum protein profiling raises the possibility of more specific diagnostic and prognostic criteria for evaluating such patients. Furthermore, the dramatic mass spectral differences observed between the sample groups led us to compare sera obtained before any treatment, from patients who ultimately differed in their response to conventional therapy. While the current work can only comment on an anecdotal basis from a limited number of these samples, some early differences were observed that suggest that a prognostic profile might exist. Beyond the obvious clinical usefulness of such a profile, it also could provide a discovery tool for further characterization of the pathophysiology of SJIA.

Although SELDI-TOF MS was recently used to compare synovial fluids from patients with rheumatoid arthritis and osteoarthritis [19], the present study is, to our knowledge, the first to define a serum proteomic profile of a rheumatic disease using SELDI-TOF MS. The SELDI-TOF MS technique described here provides a rapid, high throughput, and mass accurate method for detecting relative quantities of multiple disease-related proteins simultaneously. Using this platform, we identified a protein (SAA) known to be elevated in active SJIA. This proteomic profiling approach has the potential to expand the current repertoire of molecular targets and to provide diagnostic and prognostic information useful for improving the care of and ultimate outcome for SJIA patients.

## Conclusion

This study demonstrates the presence of serum proteomic profiles in SJIA that are reflective of active disease and suggests the feasibility of using the SELDI-TOF MS platform used as a tool for proteomic profiling in autoimmune diseases. Furthermore, the study validates the ability of the SELDI-TOF MS platform to identify a known biomarker of SJIA (SAA), suggesting that it may also be useful as a screening approach towards the discovery of novel biomarkers. To that end, identifying the 22 unknown *m*/*z *protein species in the serum profiles of our patients is now the focus of further investigation.

## Abbreviations

IL = interleukin; IMAC-3 = immobilized metal affinity capture; LC-ESI-MS/MS-TOF = liquid chromatography electrospray ionization tandem mass spectrometry time-of-flight; MALDI-TOF MS = matrix-associated laser desorption/ionization time-of-flight mass spectrometry; MRA = humanized anti-IL-6 receptor monoclonal antibody; SAA = serum amyloid A; SELDI-TOF MS = surface-enhanced laser desorption/ionization time-of-flight mass spectrometry; SJIA = systemic juvenile idiopathic arthritis.

## Competing interests

The author(s) declare that they have no competing interests.

## Authors' contributions

TM, DM, BL, and RH participated in all experimental design, data collection, and analysis and helped draft the manuscript. MM, TI, SY, and NN provided patient sera and clinical data. MW carried out the sample preparation. KK and AV carried out the LC ESI-MS/MS-TOF analysis and helped draft the manuscript. All authors read and approved the final manuscript.

## Figures and Tables

**Table 1:** SELDI-TOF MS protein peaks differentially expressed in paired sera from SJIA before and after therapy

	Before/after conventional therapy		Before/after MRA	
	
Mass (*m*/*z*)	*P*	Patients with visually distinct peaks/total no. of patients	*P*	Patients with visually distinct peaks/total no. of patients
4504	0.0001		0.005	
4758	0.0005		0.0006	
5739	0.0001		0.00000008	
6441	0.00009		0.0001	
6947	0.0005		0.003	
**9510**	**0.0007**	**6/8**	**0.00001**	**12/15**
9725	0.00006		0.00001	
**11405**	**0.00001**	**8/8**	**0.000000003**	**14/15**
**11520**	**0.000007**	**8/8**	**0.000000001**	**14/15**
**11641**	**0.000005**	**8/8**	**0.0000000001**	**14/15**
11718	0.0004		0.00002	
11880	0.0002		0.0000006	
**12703**	**0.0006**	**7/8**	**0.04**	**6/15**
**20816**	**0.0002**	**7/8**	**0.01**	**9/15**
22389	0.0002		0.00005	
23627	0.0002		0.09	
28734	0.0002		0.00006	
**33457**	**0.00001**	**6/8**	**0.00001**	**11/15**
**66637**	**0.0001**	**7/8**	**0.000009**	**11/15**
74886	0.0006		0.00003	
75622	0.0009		0.0001	
76319	0.0008		0.00002	
79216	0.0007		0.000003	

Differences between mass spectra of sera before and after conventional therapy. Sera from eight patients with SJIA who responded to conventional therapy were analyzed before and after therapy by SELDI-TOF MS using Ciphergen IMAC-3 copper chips. All samples were run in duplicate. Only variables with nonparametric *P *values of <0.001 are given. Of 282 spectral peaks identified, the 23 listed here had mean signal intensities significantly different (*P *< 0.001) before and after response to treatment. Proteins are listed according to mass/charge ratio. Visually distinct peaks (in bold type) refers to peaks that were clearly different between paired samples from before and after treatment upon visual inspection of the profiles, as shown in Fig. 1. The numbers of patients in whom these peaks were visually distinct are shown in columns 3 and 5. These peaks represent potential biomarkers of active disease. IMAC-3, immobilized metal affinity capture; MRA, humanized anti-IL-6 receptor monoclonal antibody; SELDI-TOF MS, surface-enhanced laser desorption/ionization time-of-flight mass spectrometry; SJIA, systemic juvenile idiopathic arthritis.

**Table 2:** SELDI-TOF MS unpaired serum protein peaks differentially expressed in SJIA before and after conventional therapy

Mass (*m*/*z*)	*P*
4504	0.0004
11650	0.0003
11691	0.0002
14047	0.00001
28107	0.000002
28958	0.0000005
39871	0.0004
46175	0.0008
60806	0.0002

Differences between mass spectra of pretreatment sera of 8 patients who went on to respond to conventional therapy compared with post-treatment sera of 15 patients who responded poorly to conventional therapy. Only variables with nonparametric *P *values of <0.001 are listed, from 272 peak clusters surveyed. These peaks may represent a profile predictive of response or nonresponse to conventional therapy. SELDI-TOF MS, surface-enhanced laser desorption/ionization time-of-flight mass spectrometry; SJIA, systemic juvenile idiopathic arthritis.
